# Symptomatic improvement in fibromyalgia after treatment of comorbid attention deficit hyperactivity disorder: a case report

**DOI:** 10.1186/s13256-026-05941-z

**Published:** 2026-03-18

**Authors:** Josephine Chiu, Jason Nikirk, Amayah Brown, Megan Woolford

**Affiliations:** Sam Houston State University College of Osteopathic Medicine, Conroe, TX USA

**Keywords:** Fibromyalgia, Attention deficit hyperactivity disorder, Atomoxetine, Case report

## Abstract

**Background:**

Attention deficit hyperactivity disorder is a common disorder that has an association with fibromyalgia. The connection between the two is incompletely understood but may be due to specific neurotransmitter abnormalities. Atomoxetine, a selective norepinephrine reuptake inhibitor mainly used for attention deficit hyperactivity disorder, has shown promise in relieving symptoms of attention deficit and chronic pain. However, its effectiveness in treating both conditions simultaneously has not been thoroughly studied.

**Case presentation:**

We present the case of a 33-year-old white female patient with attention deficit hyperactivity disorder and generalized pain and fatigue from suspected fibromyalgia syndrome. Investigative labs were unremarkable. Atomoxetine was prescribed as the primary treatment intervention to target attentive symptoms. Interestingly, the patient’s symptoms related to both attention deficit hyperactivity disorder and suspected fibromyalgia significantly improved within the first 2 weeks, with continuous relief over the next 9 months. The patient also reported improved quality of mood, sleep, and tolerance of physical activity.

**Conclusion:**

This case of a 33-year-old female with attention deficit hyperactivity disorder and chronic pain suspicious for fibromyalgia showed the improvement of pain symptoms with atomoxetine. While a few published cases have demonstrated a similar finding, this case is notable for the dramatic improvement and the response to a nonstimulant medication—atomoxetine. This case highlights the need for further research into the connection between attention deficit hyperactivity disorder and chronic pain, as well as the mechanism behind the effects of atomoxetine on pain.

## Background

Attention deficit hyperactivity disorder (ADHD) is a common neurodevelopmental disorder with onset in childhood but persists into adulthood in some patients. According to the Diagnostic and Statistical Manual of Mental Disorders, Fifth Edition (DSM-5), symptoms characterizing inattention or hyperactivity must have started before age 12 years and lasted at least 6 months [1]. Stimulant medications (such as methylphenidate or amphetamines) and nonstimulant medications (such as atomoxetine, clonidine, or guanfacine) are commonly prescribed. These medications improve regulation of dopamine and norepinephrine activity in the central nervous system, thus improving attention, impulse control, and hyperactivity [2].

Fibromyalgia is a chronic pain disorder characterized by widespread musculoskeletal pain, fatigue, insomnia, and cognitive difficulties that may dramatically reduce the patient’s quality of life. After rigorous exclusion of alternative diagnoses, the presence of fibromyalgia can be evaluated with the diagnostic criteria established by the American College of Rheumatology [3]. The Food and Drug Administration (FDA)-approved medications to treat fibromyalgia include serotonin–norepinephrine reuptake inhibitors (SNRIs) duloxetine and milnacipran and the anticonvulsant GABA analog pregabalin.

ADHD-type symptoms have been reported in 25–32% of patients with fibromyalgia [4, 5]. Interestingly, some medications for ADHD have reduced chronic pain in select case reports [6, 7]. This case further adds to these reports and highlights the need for further studies investigating the connection between ADHD and chronic pain, specifically fibromyalgia.

## Case presentation

A 33-year-old white female patient presented to the clinic in April 2020 with symptoms of severe fatigue, reduced exercise tolerance, insomnia, and chronic musculoskeletal pain with greatest intensity in the hips and lower back. Her fatigue and chronic pain had been present for more than 5 years. She was an avid runner prior to the onset of symptoms, but her fatigue progressively limited any vigorous physical activity. Past medical history on initial visit included a partial hysterectomy at age 32 years for abnormal uterine bleeding, mild intermittent asthma, and multiple environmental allergies. The patient also reported consumption of roughly 550 mg of caffeine daily in the form of coffee and espresso. She denied nicotine, alcohol, or substance use.

At later visits, the patient described fluctuations in body temperature, running as low as 93 °F with spikes of up to 99 °F. These episodes correlated with symptoms of facial flushing, blurred vision, and chest palpitations followed by an abrupt onset of headache. However, her temperature at all office visits fell within the normal range.

Careful physical examination revealed breast tenderness, spasms of the paraspinal and trapezius muscles bilaterally, and anxious mood over the span of the first five visits. Laboratory workup, including complete blood count (CBC), comprehensive metabolic panel (CMP), C-reactive protein (CRP), and erythrocyte sedimentation rate (ESR) were within normal limits. Antinuclear antibodies (ANA) and rheumatoid factor (RF) labs were negative. Vitamin D3, B12, and folate levels were normal. Cortisol, follicular stimulating hormone (FSH), luteinizing hormone (LH), and thyroid-stimulating hormone (TSH) levels were normal.

Given the lack of physical exam findings of other diseases and normal laboratory workup, fibromyalgia was suspected as the source of her fatigue and chronic pain. Systemic lupus erythematosus and rheumatoid arthritis were considered less likely owing to a lack of characteristic findings on physical exam and were ultimately ruled out using antinuclear antibody and rheumatoid factor tests, respectively. Given the cyclical pattern of flushing and temperature fluctuations, primary ovarian insufficiency was suspected; however, the normal hormone levels, as mentioned above, essentially ruled out this disorder. According to the American College of Rheumatology (ACR) criteria 2016 revision, a diagnosis of fibromyalgia would require a Widespread Pain Index (WPI) of 7 or greater with Symptom Severity Scale of 5 or greater [8]. Though our patient’s scores did not reach these diagnostic thresholds, her presentation was suspicious for fibromyalgia given the chronic pain, headaches, fatigue and sleep disturbance.

To rule out contributions of excessive caffeine intake [9], she was advised to reduce her daily consumption from 550 mg to less than 100 mg. Chronic musculoskeletal pain and fatigue did not improve, and she reported severe ADHD symptoms, predominantly inattentiveness and inability to complete tasks both at home and at her workplace. The patient reported a history of inattentive-type symptoms since early childhood but was never formally diagnosed with ADHD. She described difficulties with school due to excessive talking and lack of attention, even resulting in accidental injury. We hypothesize that the extremely high caffeine intake was a coping mechanism to alleviate the symptoms of ADHD. High caffeine intake has been associated with reduced ADHD symptoms among US Army soldiers with adult ADHD [10].

According to DSM-5 criteria, this patient was diagnosed with ADHD predominantly inattentive type. Because the symptoms were severe enough that she was at risk of losing her employment, atomoxetine was prescribed to treat her ADHD at a dose of 25 mg daily (November 2020) and increased to 25 mg twice daily within a week. The dose was further increased to 40 mg twice daily (December 2020) but eventually reduced back to 25 mg twice daily (April 2021) owing to complaints of brain fog.

Within 3 weeks of starting atomoxetine, the patient noticed drastic improvements in her ADHD symptoms. Surprisingly, the patient also reported substantial improvements in her fatigue, chronic pain, and insomnia. Within 2 months, the patient reported a near-complete resolution of the fatigue, musculoskeletal pain, and insomnia, which had been present for more than 5 years. She was also able to resume her previous lifestyle activities, including running. All improvements were sustained for 9 months of follow-up. The patient did report stopping atomoxetine on two separate occasions afterward, owing to a desire to reduce overall medication usage, but resumed taking her prescription when ADHD symptoms quickly returned. She eventually discontinued atomoxetine in February of 2023 (28 months later) owing to a side effect of blurred vision. Remarkably, even after discontinuing atomoxetine, the patient reported a sustained reduction in chronic pain compared with her initial visit.

## Discussion

While fibromyalgia and ADHD are distinct disorders with different symptomatic profiles, they may share pathophysiologic mechanisms. Several studies describe an association between ADHD and fibromyalgia, [11, 12]. One study discovered that patients with attention deficit disorders were more sensitive to pain than the control group, suggesting a physiological link between attention deficit and pain. Interestingly, in that study, the sensitivity was partially reversed by treatment with methylphenidate [13]. The mechanisms underlying central sensitization in these conditions are not fully understood but may involve dysregulation of neurotransmitters and altered pain processing pathways [14]. In fibromyalgia, this manifests as increased pain perception that may be influenced by attentional biases toward painful stimuli, while in ADHD, it may manifest as hypersensitivity to sensory stimuli, including pain, or reduced pain tolerance.

Both stimulant medications and NRIs have some ability to reduce symptoms of fibromyalgia. However, NRIs such as atomoxetine have a few favorable characteristics over stimulants, including little to no abuse potential, decreased regulatory hurdles, and fewer medication interactions. In some patients, atomoxetine appears to have a superior effect on chronic pain than stimulants [6, 7].

Several confounders in this patient’s presentation include excess caffeine use, concurrent sleep disturbance, and a sedentary lifestyle. However, on the basis of the temporal association between the improvement of her symptoms and the start of atomoxetine, the authors believe that these confounders are less significant. In terms of her caffeine use, after she was instructed to reduce caffeine, her pain, fatigue, and difficulty sleeping still persisted. It was only after starting atomoxetine that these improved, along with improvement of the worsened inattentive symptoms. The onset of fatigue preceded her heavy caffeine use by several years. Primary sleep disorders such as insomnia can certainly contribute to fatigue and pain perception [15]; however, her treatment plan did not include pharmacologic therapy for insomnia at the time of her improvement. She was instructed in sleep hygiene practices, so it is possible that these improved her sleep and partly contributed to the improvement in her symptoms. Sedentary lifestyle is known to be associated with fatigue [16], which cannot be entirely ruled out as a confounder for this patient. She did however report being an avid runner prior to the onset of her symptoms, with fatigue limiting her ability to participate in her hobby. Notably, after atomoxetine was started, she was able to resume physical activity.

This case report illustrates a possible shared mechanism between ADHD and fibromyalgia, but is limited as only one patient was observed. Given the single sample, it is difficult to draw reliable conclusions regarding the association between her symptomatic improvement and new medication. In addition, the symptoms that improve—pain, fatigue, and attentiveness—are subjectively reported, and standardized scales are not readily available for fatigue and attentiveness. This makes it impossible to quantify the size of the improvement effect. An additional limitation is the discontinuation of the medication by the patient owing to adverse effects.

Despite the limitations of this study, the findings suggest further studies with higher patient numbers are warranted. Fibromyalgia is a chronic debilitating disease that is poorly understood, with limited pharmacologic treatment options. Expanding the options, especially with medications that may treat a common comorbidity as well, would improve quality of life for patients with this painful condition. In addition, several of the other commonly used medications have undesirable side effects, most notably dizziness, drowsiness, and weight gain. Atomoxetine may be better tolerated, especially in adult patients.

## Conclusion

The improvement in pain and fatigue our patient experienced on atomoxetine suggests that it may be a dual therapeutic option in patients with fibromyalgia and ADHD. Should further study show its effectiveness, clinicians could consider using atomoxetine preferentially for ADHD symptoms to obtain improvement in both conditions. While the exact mechanism by which atomoxetine appears to improve pain is not known, it is a promising research target, as illustrated by our patient and other cases in literature. Further research into how atomoxetine affects pain signaling is warranted.

## Data Availability

Data sharing is not applicable since no datasets were generated or analyzed in this study.

## Abbreviations

ADHD: Attention deficit hyperactivity disorder
ANA: Antinuclear antibody
CBC: Complete blood count
CMP: Comprehensive metabolic panel
CRP: C-reactive protein
DSM-5: Diagnostic and Statistical Manual of Mental Disorders, Fifth Edition
ESR: Erythrocyte sedimentation rate
FSH: Follicle-stimulating hormone
LH: Luteinizing hormone
RF: Rheumatoid factor
SNRI: Serotonin–norepinephrine reuptake inhibitor
TSH: Thyroid-stimulating hormone
WPI: Widespread Pain Index
